# Prostate Cancer-Associated Disseminated Intravascular Coagulation with Excessive Fibrinolysis Treated with Degarelix

**DOI:** 10.1155/2015/212543

**Published:** 2015-11-03

**Authors:** Shawn Y. Ong, Josephine Taverna, Clint Jokerst, Thomas Enzler, Emad Hammode, Elisa Rogowitz, Myke R. Green, Hani M. Babiker

**Affiliations:** ^1^University of Arizona College of Medicine, 1501 N. Campbell Avenue, Tucson, AZ 85724, USA; ^2^University of Arizona Cancer Center, 1515 N Campbell Avenue, Tucson, AZ 85724, USA; ^3^Department of Radiology, University of Arizona College of Medicine, 1501 N. Campbell Avenue, Tucson, AZ 85724, USA; ^4^Department of Medicine, University of Arizona College of Medicine, 1501 N. Campbell Avenue, RM 6336, Tucson, AZ 85724, USA; ^5^Scripps Health, Department of Medicine, 10666 N Torrey Pines Road, La Jolla, CA 92037, USA; ^6^Department of Pharmacy Services, University of Arizona Medical Center, 1501 N Campbell Avenue, Tucson, AZ 85724, USA; ^7^Virginia G. Piper Cancer Center Clinical Trials at HonorHealth Research Institute, 10510 N 92nd Street, Suite 200, Scottsdale, AZ 85258, USA

## Abstract

Disseminated intravascular coagulation (DIC) with excessive fibrinolysis (XFL) is a rare and acute life-threatening variant of DIC in patients with prostate cancer. Patients present with coagulopathy, hypofibrinogenemia, and systemic bleeding. We describe a case of DIC XFL caused by prostate cancer (PC) successfully treated with a single injection of degarelix, a gonadotropin-releasing hormone (GnRH) receptor antagonist. This led to prompt control of the patient's coagulopathy within ten days of treatment. Our case highlights features of this rare and devastating hemorrhagic complication of PC along with a fast-acting and effective therapeutic drug option.

## 1. Case Description

A 61-year-old Hispanic man with past medical history of benign prostatic hyperplasia (BPH) treated with transurethral resection of the prostate the preceding year presented to our emergency room with a nonhealing wound on his right thigh. He reported that he sustained a laceration to his right thigh during work with machinery one week prior to presentation and had visited the nearest urgent care. Despite suture placement at the urgent care, he continued to bleed. In addition, he reported multiple ecchymoses on his lower abdomen and extremities and severe pain in his left hip and leg. The patient had a temperature of 36.5°C, a heart rate of 101 beats per minute, a blood pressure of 138/87 mm Hg, a respiratory rate of 18 breaths per minute, and an oxygen saturation of 98% on room air. Physical exam revealed skin pallor along with diffuse ecchymoses of the abdomen, thighs, and extremities. No lymphadenopathy was noted. Labs revealed a WBC of 7.9 (normal 3.4–10.4 1000/*μ*L), hemoglobin 8.2 (normal 13.5–17.5 g/dL), hematocrit 24.8 (normal 40.0–51.0%), platelet counts of 76 (normal 150–425 1000/*μ*L), INR 1.8 (normal 0.8–1.1), PT 20.9 (normal 11.9–15.0 seconds), PTT 46.9 (normal 22.6–35.5 seconds), fibrinogen 40 (normal 200–430 mg/dL), alkaline phosphatase 1193 (normal 40–150 IU/L), and D-dimer >20.0 (normal <0.5 *μ*g/mL). The patient was started on 4 units of fresh frozen plasma and 1 unit of cryoprecipitate, and a DIC panel was obtained every 6 hours.

**Figure 1:**
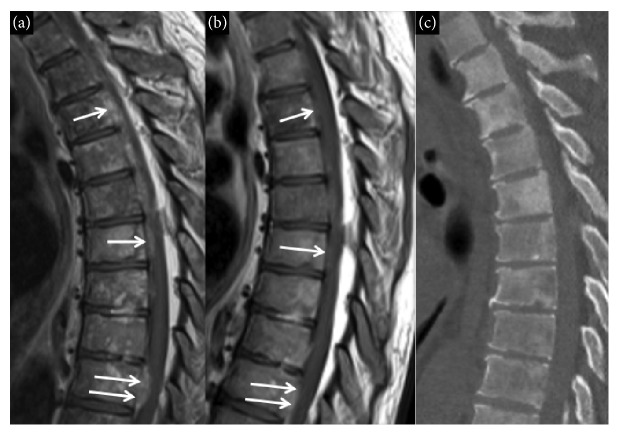
Sagittal contrast-enhanced T1-weighted image of the thoracic spine (a) demonstrates multiple deposits of enhancing soft tissue in the ventral aspect of the epidural space (arrows) consistent with epidural metastatic disease in this patient with prostate cancer. Also note the heterogeneous bone marrow signal consistent with diffuse osseous metastatic disease. A follow-up study obtained just 39 days later (b) demonstrates resolution of epidural metastasis (arrows). Sagittal reconstruction from a thoracic spine CT (c) demonstrates patchy sclerosis in the bone consistent with diffuse osseous metastatic disease.

**Figure 2:**
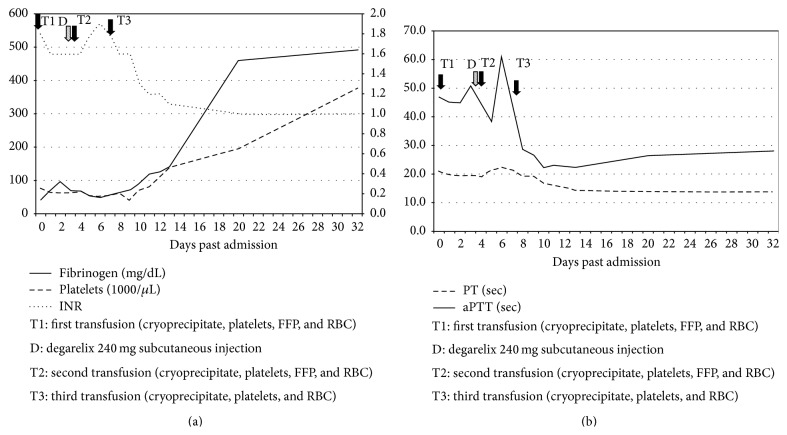
Graph (a) demonstrates the time of administration of degarelix and fibrinogen, platelets, and INR trends over the course of hospitalization. (b) Graph demonstrates PT and PTT trends over the course of hospitalization.

An X-ray of the left hip and femur demonstrated extensive metastatic lesions of the left ilium, ischium, and pubic bones including involvement of the acetabulum. A contrast-enhanced chest CT revealed focal periosteal reaction in the left posteromedial 6th, 7th, and 8th ribs along with nonhomogenous mixed sclerotic and lytic appearance in the spine. An abdominal MRI with and without contrast demonstrated diffuse osseous metastatic disease but no evidence of primary malignancy in the abdomen. Nuclear medicine whole body bone scan revealed diffuse osseous metastatic disease involving the axial and proximal appendicular skeleton along with bilateral involvement of the femurs. Spinal MRI with and without contrast demonstrated diffuse osseous metastatic disease involving the cervical, thoracic, lumbar spine, and sacrum. The MRI also demonstrated an unusual finding of extensive metastatic epidural tumor deposits along the thoracic and lumbosacral spine resulting in spinal canal stenosis (Figure 1). The patient required transfusions of cryoprecipitate (CP), fresh frozen plasma (FFP), platelets (PLTs), and red blood cells (PRBCs) pending workup (Figures 2(a) and 2(b)). Prostate serum antigen (PSA) and *α*2-antiplasmin (AAP) levels were ordered and revealed PSA and AAP levels of 673.17 (normal 0.0–4.0 ng/mL) and 42 (normal 88–120%), respectively. The significantly decreased fibrinogen and AAP levels in conjunction with high PSA indicated PC with DIC XFL as the diagnosis. A prostate biopsy was not attempted given the high risk of bleeding. The patient received two subcutaneous injections of degarelix 120 mg on each side of his abdomen (total 240 mg), followed immediately by cold compresses to both areas to prevent bleeding. Over the next 7 days, he was transfused with CP, PRBCs, PLTs, and FFP. He also received Vitamin K 5 mg daily. Coagulopathy marker goals were fibrinogen level > 100 mg/dL, hemoglobin > 8 g/dL, and platelet count > 50,000/*μ*L. Ten days after admission, his coagulopathy improved obviating the need for transfusions (Figures 2(a) and 1(b)).

The patient was observed an additional two days and did not require supportive transfusions and was discharged on day 13 after admission. Over the course of his encounter, he received a total of 70 units of CP, 8 units of FFP, 4 units of PRBCs, and 3 units of PLTs. His lab values upon discharge were platelets 139,000/*μ*L, INR 1.1, PT 14.4 sec, PTT 22.4 sec, fibrinogen 141 mg/dL, alkaline phosphatase 600 IU/L, and PSA 125.70 ng/mL.

During an outpatient followup, the patient was stable and his labs revealed no coagulopathy and his D-dimer remained persistently elevated. He decided against pursuing a prostate biopsy given his concerns of bleeding and low likelihood that results will change management. MRI of the spine one month after starting testicular suppression revealed complete resolution of the epidural deposits (Figure 1). After 3 months of treatment with degarelix his PSA decreased to 26 and he achieved a very good partial response by imaging. Six months after starting hormonal therapy he developed urinary retention requiring hospitalization and urinary catheter placement. His PSA increased to 555 and a bone scan and an MRI of the spine, abdomen, and pelvis revealed extensive bone metastasis in thoracic and lumbar spine, femur, ribs, and pelvis. Bicalutamide was added; however, his disease progressed with a PSA level of 1361 and an alkaline phosphatase level of 1247 (normal 40–150 IU/L). He was started on docetaxel and received 3 cycles of therapy. His PSA remained elevated at 1200 and his performance status worsened significantly (Karnofsky performance status of 60%) necessitating admission to a rehab center. The patient shortly after decided on comfort care.

## 2. Discussion

PC is the most frequently diagnosed nonskin malignancy in men and the third leading cause of cancer deaths in the USA [1]. Patients can present with various disease manifestations but localized lower urinary tract symptoms that include urinary retention, frequency, hesitancy, nocturia, and hematuria are the most common complaints [2]. Hematological disorders associated with PC include thrombotic thrombocytopenia, primary fibrinolysis, sterile thrombotic endocarditis, and thrombosis [3]. DIC, however, is the most common hematological ailment observed in PC patients (13–30%) [3, 4]. The exact pathophysiology underlying the association between PC and DIC is partially understood [5]. Previous reports indicated that tumor cells express a variety of procoagulant molecules, including tissue factor (TF), which activates the host's hemostatic system leading to thrombosis and consumption of the coagulation factors culminating to DIC [6]. Recent data further elucidated this hypothesis by identifying the release of microparticles (MP) from tumor cells (membrane blebs that are released from the cell surface) as the main culprit stimulating the coagulation cascade [7]. MP provide a membrane surface for the assembly of the components of the coagulation protease cascade. In addition, the presence of anionic phospholipids (phosphatidylserine) and TF in MP increases the procoagulant activity through activation of factor VII and the extrinsic coagulation cascade leading to DIC [7]. Patients present with elevated PT, PTT, INR, D-dimer, fibrin or fibrinogen degradation products, thrombin-antithrombin complex, positive fibrin monomer test, low PLTs, and normal or near normal fibrinogen levels (indicating that fibrinolysis is compensated) [5, 8]. Patients with PC and DIC with underlying compensated fibrinolysis rarely present with bleeding (0.4 to 1.65%) and tend to develop thrombotic rather than hemorrhagic complications [5, 8]. Conversely, patients with PC and XFL usually develop a unique and life-threatening form of DIC manifested by systemic intracavitary, intracutaneous, and intracranial bleeding [5]. This is secondary to hypofibrinogenemia due to uncompensated XFL that might result from the overproduction of urinary-type plasminogen activators (uPA) by prostate cancer cells or the depletion of fibrinolytic inhibitors such as AAP [9, 10]. Plasmin activity is regulated by the enzyme AAP, which inactivates plasmin and limits its actions. When the level of AAP falls below 60% of normal then plasmin is able to degrade fibrinogen, thus resulting in XFL and a bleeding diathesis [10]. PC cells produce an excess amount of uPA that increase the amount of circulating plasmin [11].

In the largest retrospective study of PC with DIC and XFL (defined as hypofibrinogenemia, coagulopathy, decreased AAP, thrombocytopenia, and hemorrhage and/or thrombosis) patients had advanced disease on presentation [5]. The study identified forty-two PC patients with XFL and revealed that most patients on presentation had metastatic disease (95%), high-grade tumors (>50% with Gleason scores >7), and castration-resistance disease (93%), received at least two lines of therapy (90%), and received previous treatment with taxanes (50%) [5]. Laboratory evaluation indicated elevated D-dimers and PT, thrombocytopenia, low AAP, and a normal PTT range for the majority of patients. 79% of the patients developed some bleeding complication that was intracutaneous (33%), genitourinary (26%), gastrointestinal (18%), and intracranial (28%). The report indicated a median survival of four weeks and reversal of DIC with XFL occurred in only 18% of patients, all of whom received chemotherapy in addition to supportive therapy with transfusions. This cohort of patients had an improved median survival of 26 weeks in comparison to patients who did not achieve a DIC and XFL reversal, highlighting the importance of both prompt recognition and treatment. Similar to the aforementioned survival findings our patient had poor prognosis and succumbed to his illness almost one year since diagnosis.

A case report of a 51-year-old male who presented with extensive purpura and anemia was admitted to the hospital and labs revealed elevated PT, PTT, thrombin-antithrombin complex, fibrin degradation products (FDPs), D-dimers, plasmin-antiplasmin complex (PAP), normal PLTs, positive fibrin monomer test, and low AAP [8]. Immunohistochemical stains of biopsied prostate cancer cells were positive for TF and uPA confirming PC as the cause of DIC with XFL. Treatment with low-molecular-weight heparin (LMWH) failed to improve fibrinolytic markers by increasing fibrinogen, D-dimers/FDP ratio, and AAP or decreasing PAP. Lack of improvement with anticoagulation suggested a stronger primary fibrinolytic drive. With the addition of tranexamic acid, the authors reported an increase in AAP and fibrinogen and improvement in the remaining fibrinolytic markers corroborating the premise of uncompensated XFL drive. Common distinctive laboratory findings between DIC and XFL are depicted in Table 1. Our patient's laboratory work indicated low fibrinogen and AAP and coagulopathy alluding to a state of DIC with XFL. One of the limitations of our case report is the lack of histological confirmation. A biopsy was not attempted during hospitalization due to significant risk of bleeding and during outpatient followup the patient declined a biopsy in fear of recurrent bleeding. Another limitation is that a repeat AAP and baseline fibrinogen-degradation products were not obtained.

Many different therapies have been applied to patients who develop PC with DIC and XFL. Cooper et al. reported successfully using epsilon-aminocaproic and low-dose heparin to treat a patient with DIC with XFL [12]. Leong et al. attempted nuclear medicine with Strontium-89 but it failed at controlling bleeding and in fact worsened coagulopathy [13]. Review of the literature highlighted success with ketoconazole, a drug that can achieve castrate levels of testosterone within 48 hours and does not cause an androgen surge seen with GnRH analogues [14, 15]. Our patient was not treated with ketoconazole due to QT prolongation on EKG. Initiation of GnRH agonists alone in the treatment of PC with XFL has been avoided due to concerns that an androgen surge can deteriorate coagulopathy and lead to other complications, such as cord compression in our patient due to the epidural deposits [16]. The benefit of combining ketoconazole to other hormonal therapies to increase efficacy is unclear [17]. Other hormonal treatment strategies attempted include estrogen analogues; however, this is not recommended given the concern of worsening coagulation and other reports implicating worsening DIC and XFL in some cases [18–21]. Other successful treatment strategies reported included treatment with combined LMWH, tranexamic acid, and high dose antiandrogen therapy [8]. Treatment with bicalutamide and leuprolide has also been successful [3]. The standard of care has been treatment with chemotherapy reserved for patients not responding to hormonal therapies or have castrate-resistant disease. However, a recent trial enrolled 790 patients with metastatic hormone-sensitive disease and randomized them to androgen deprivation therapy (ADT) with or without docetaxel (75 mg/m^2^ every 3 weeks for 6 cycles) [22]. The results of the trial revealed better cancer control, longer time to the development of castration resistance, higher rate of decrease of PSA level, lower number of prostate-cancer deaths, and substantially longer overall survival in the combination group. Maximum benefit with a median overall survival of 17 months was derived in patients with high-volume disease defined as presence of visceral metastasis or four or more bony lesions with at least one lesion beyond the vertebral bodies or pelvis. Another trial highlighted a survival benefit to the addition of docetaxel but not zoledronic acid to hormonal therapy in patients with high risk locally advanced or metastatic prostate cancer [23]. Conversely, there are no data on the effectiveness of either of those regimens in patients with PC and XFL specifically.

A variety of cytotoxic therapies including mitoxantrone/prednisone, docetaxel and cisplatin, and single agent docetaxel have been tried with some success in patients with PC and XFL [5, 24, 25]. Degarelix is a third generation LHRH antagonist used in men with prostate carcinoma and where hormonal treatment is indicated [26]. This drug antagonizes pituitary GnRH receptors thus suppressing gonadotropin (LH and FSH) release leading to reduction in testicular steroidogenesis. It was approved by the US Food and Drug Administration in 2008 for androgen-dependent prostate cancer [26]. Data from a Phase III clinical trial that led to its approval demonstrated that degarelix achieved faster PSA suppression and no testosterone surge compared to leuprolide [27]. The clinical trial revealed testosterone suppression in a vast majority of patients after 3 days compared to leuprolide at 14 days. This advantageous medication profile was particularly important for our patient since his tumor burden was directly related to the extensive fibrinolysis and a delay in response or an increase in testosterone would likely have worsened his condition. To the best of our knowledge this is the first reported case of successful treatment of DIC with XFL with a single injection of degarelix.

## 3. Conclusion

PC with XFL is a distinctive hematological complication associated with life-threatening bleeding, advanced disease at presentation, high-grade and castrate-resistant disease, and poor prognosis. Prompt diagnosis in patients presenting with idiopathic coagulopathy and fibrinolysis is paramount and early treatment is crucial to prevent mortality due to acute severe bleeding. Supportive therapy with transfusions should be started immediately and early PC directed therapy with antiandrogens such as degarelix, ketoconazole, or chemotherapy can be life saving for patients with PC and XFL.

## Figures and Tables

**Table 1:** Distinctive laboratory parameters in DIC and XFL.

	DIC	XFL
Elevated	(1) PTT. (2) PT. (3) INR. (4) D-dimers. (5) TAT.	(1) FDP. (2) PAP.

Decreased	(1) PLTs. (2) Fibrinogen *(normal to mildly decreased)*	(1) Fibrinogen. (2) AAP. (3) D-dimers/FDP ratio.

Positive	Positive fibrin monomer test.	Positive fibrin monomer and uPA tests.

AAP: alpha 2 antiplasmin; DIC: disseminated intravascular coagulation; FDP: fibrin degradation product; INR: international normalized ratio; PAP: plasmin-antiplasmin complex; PLTs: platelets; PT: prothrombin time; PTT: partial thromboplastin time; TAT: thrombin-antithrombin complex; uPA: urinary-type plasminogen activators; XFL: excessive fibrinolysis.
